# Mercury is present in neurons and oligodendrocytes in regions of the brain affected by Parkinson’s disease and co-localises with Lewy bodies

**DOI:** 10.1371/journal.pone.0262464

**Published:** 2022-01-11

**Authors:** Roger Pamphlett, David P. Bishop

**Affiliations:** 1 Sydney Medical School, Brain and Mind Centre, The University of Sydney, Sydney, New South Wales, Australia; 2 Department of Neuropathology, Royal Prince Alfred Hospital, Sydney, New South Wales, Australia; 3 Elemental Bio-Imaging Facility, School of Mathematical and Physical Sciences, University of Technology Sydney, Sydney, New South Wales, Australia; Indiana University, UNITED STATES

## Abstract

**Objective:**

Environmental toxicants are suspected to play a part in the pathogenesis of idiopathic Parkinson’s disease (PD) and may underlie its increasing incidence. Mercury exposure in humans is common and is increasing due to accelerating levels of atmospheric mercury, and mercury damages cells via oxidative stress, cell membrane damage, and autoimmunity, mechanisms suspected in the pathogenesis of PD. We therefore compared the cellular distribution of mercury in the tissues of people with and without PD who had evidence of previous mercury exposure by mercury being present in their locus ceruleus neurons.

**Materials and methods:**

Paraffin sections from the brain and general organs of two people with PD, two people without PD with a history of mercury exposure, and ten people without PD or known mercury exposure, were stained for inorganic mercury using autometallography, combined with immunostaining for a-synuclein and glial cells. All had mercury-containing neurons in locus ceruleus neurons. Laser ablation-inductively coupled plasma-mass spectrometry (LA-ICP-MS) was used to confirm the presence of mercury and to look for other potentially toxic elements. Autometallography-stained locus ceruleus paraffin sections were examined to compare the frequency of previous mercury exposure between 20 PD and 40 non-PD individuals.

**Results:**

In PD brains, autometallography-detected mercury was seen in neurons affected by the disease, such as those in the substantia nigra, motor cortex, striatum, thalamus, and cerebellum. Mercury was seen in oligodendrocytes in white and grey matter. Mercury often co-localised with Lewy bodies and neurites. A more restricted distribution of brain mercury was seen in people without PD (both with or without known mercury exposure), with no mercury present in the substantia nigra, striatum, or thalamus. The presence of autometallography-detected mercury in PD was confirmed with LA-ICP-MS, which demonstrated other potentially toxic metals in the locus ceruleus and high iron levels in white matter. Autometallography-detected mercury was found in locus ceruleus neurons in a similar proportion of PD (65%) and non-PD (63%) individuals.

**Conclusions:**

In people with PD, mercury was found in neurons and oligodendrocytes in regions of the brain that are affected by the disease, and often co-localised with aggregated a-synuclein. Mercury in the motor cortex, thalamus and striatum could result in bradykinesia and rigidity, and mercury in the cerebellum could cause tremor. People without PD had a restricted uptake of mercury into the brain. The similar frequency of mercury in the locus ceruleus of people with and without PD suggests these two groups have had comparable previous mercury exposures but that PD brains have a greater predisposition to take up circulating mercury. While this post mortem study does not provide a direct link between mercury and idiopathic PD, it adds to the body of evidence that metal toxicants such as mercury play a role in the disease. A precautionary approach would be to reduce rising mercury levels in the atmosphere by limiting the burning of fossil fuels, which may be contributing to the increasing incidence of PD.

## Introduction

Parkinson’s disease (PD) is a common neurodegenerative condition with a large socioeconomic burden [1]. Clinical and pathological features of PD include a world-wide distribution, male predominance, a variable clinical presentation and progress, motor, non-motor and multisystem clinical involvement, a long prodromal period during which constipation and sleep disorders are common, pathogenic pathways involving oxidative stress, inflammation, and damage to membrane systems such as mitochondria and lysosomes, and collections of aggregated a-synuclein in Lewy bodies and neurites [1]. The incidence and prevalence of PD have risen markedly in the past two decades [1]. This growth is not all attributable to population aging, and we appear to be facing a Parkinson’s pandemic [2].

Single gene abnormalities and genetic variants with low individual effect sizes are responsible for only a minority of later-onset PD cases [1]. The reported increased risk of PD in people with an affected first-degree relative could indicate a shared environment, with twin studies showing a similar concordance in monozygotic and dizygotic twins, and 90% of people with PD have no family history of PD [3]. Attention has therefore turned to environmental factors that could trigger PD, possibly via gene-environment interactions [3–6]. Epidemiological studies, some supported by animal and in vitro models of PD pathology, have implicated potentially toxic substances including pesticides, solvents, and metals such as iron and mercury as possible pathogenic agents [3]. However, it is difficult to estimate toxicant exposures from epidemiological data, especially for diseases that may start in early life. In addition, bulk chemical analyses of tissues affected by PD are not sensitive enough to detect toxicants that affect only a small proportion of cells. Therefore, elemental bioimaging of potentially toxic elements at the cellular level is needed to estimate the toxicant burden in the brains of people with neurodegenerative disorders [7].

Several indirect lines of evidence suggest that an environmental toxicant such as mercury could play a role in PD. The steadily increasing amount of mercury vapor in the atmosphere [8], and consequently in fish [9], could underlie the increased incidence of PD [2]. Atmospheric mercury is distributed world-wide via the atmosphere-water-soil cycle [10, 11], which accords with the global prevalence of PD [12]. Men are more likely to be exposed to mercury in industrial occupations, and men are more at risk of getting PD [1]. The uptake of mercury in humans is variable and depends on the chemical form of mercury and source of exposure [13, 14], which fits with the variation in PD symptoms between patients [1]. An early-life exposure to mercury, for example from eating mercury-contaminated fish, could seed the brain and other organs with methylmercury, with later cell damage due to the slow demethylation of methylmercury to inorganic mercury [15], which would result in PD motor symptoms manifesting only later in life [1]. The prevalence of mercury in human cells increases with aging [16], which could underlie the symptoms of PD increasing in later ages. Mercury has multiple toxic effects which include the generation of free radicals [17], autoimmune inflammation [18], and attachment to sulfhydryl-rich cell membranes in organelles such as mitochondria, lysosomes, and the Golgi apparatus [13, 19], all features implicated in the pathogenesis of PD [1]. Finally, mercury can induce a-synuclein aggregation [20] and so could be involved in the generation of Lewy bodies and neurites.

To look for evidence that mercury could underlie some pathogenetic aspects of PD, we used two elemental bioimaging techniques to study the distribution of mercury in people with and without PD. To look for differences in mercury uptake, we chose to study people with evidence of previous mercury exposure based on their having mercury in locus ceruleus neurons, where mercury persists for long periods of time [7]. We studied tissue from forensic/coronial autopsies, since people who die unexpectedly or from unnatural causes are more likely to have earlier stages of PD, which makes identifying causative toxicants more feasible. Furthermore, in forensic autopsies non-central nervous system (CNS) organs are sampled so any role mercury might play in these organs in PD [21–23] could be assessed.

## Materials and methods

### Ethics

This study (X14-029) was approved by the Human Research Committee, Sydney Local Health District (Royal Prince Alfred Hospital Zone). This institutional review board waived the need for written informed consent from relatives of individuals studied since this was a de-identified retrospective study of archived paraffin-embedded tissue. Data were fully anonymised on the research database after initial access to Department of Forensic Medicine records.

### Sample collection

#### Multifocal brain samples

Paraffin-embedded tissue blocks from multifocal locations within the cerebrum, cerebellum and brain stem were obtained from the tissue archive of The New South Wales Department of Forensic Medicine from 14 people who had evidence of previous exposure to mercury because their locus ceruleus neurons in the rostral pons had been found in a previous study to contain mercury [7]. The samples studied were from: (**1**) Two women with a clinical diagnosis of idiopathic PD and neuropathological evidence of Lewy body-positive PD, aged 72 and 76 years (PD1 and PD2), (**2**) Two men with known sources of mercury exposure, one aged 24 years who had been exposed to self-injected metallic mercury for five months (ME1) [24], and one a professional fisherman aged 39 years who was probably exposed to methylmercury from seafood consumption (ME2) [25], and (**3**) Ten people without PD and without known sources of mercury exposure, aged 59–104 years, 3 male and 7 female.

#### Locus ceruleus-only samples

To compare the frequency of mercury exposure of people with and without PD, paraffin sections of rostral pons containing the locus ceruleus that had been stained with autometallography in a previous study [7] were re-examined. These were from (**1**) 20 PD patients (10 male, 10 female, mean age 74 years, SD 9 years, age range 59–95 years), including the two PD patients above, and (**2**) 40 non-PD individuals (20 male, 20 female, mean age 78 years, SD 14 years, age range 59–95 years, consisting of 20 with non-PD neurodegenerative disorders (15 with Alzheimer’s disease, 2 with multiple system atrophy, and one each with frontotemporal dementia, progressive supranuclear palsy, and myotonic dystrophy), 15 with no major pre-mortem conditions, 4 with a psychosis (2 with bipolar disorder, and 1 each with depression and schizophrenia), and 1 with cancer. The frequencies of the presence of autometallography-detected iHg in locus ceruleus neurons in these two groups were compared with contingency analysis and Fisher’s exact test using Prism 9 software.

### Autometallography

Paraffin blocks were sectioned at 7 μm with a Feather S35 stainless steel disposable microtome blade, deparaffinised, and stained with silver nitrate autometallography, which represents the presence of inorganic mercury (iHg) as black silver grains surrounding the mercury [26]. Autometallography is a sensitive amplification technique that can detect as few as 10 mercury sulphide/selenide molecules in a cell [27]. Sections were placed in physical developer containing 50% gum arabic, citrate buffer, hydroquinone and silver nitrate at 26°C for 80 minutes in the dark, washed in 5% sodium thiosulphate to remove unbound silver, counterstained with mercury-free hematoxylin or Luxol-fast blue, and viewed with bright-field microscopy. Sections were stained with hematoxylin only to act as a control for the autometallography. Each staining run included a control section of mouse spinal cord where motor neuron cell bodies contained mercury following an intraperitoneal injection of mercuric chloride; these sections were from archived paraffin blocks of a previously-published experiment approved by the Animal Ethics Committee of the University of Sydney [28]. Representative sections were stained with autometallography and then immunostained for a-synuclein with 1:500 monoclonal mouse-anti-human alpha-synuclein (Invitrogen 328100), astrocytes with 1:2000 polyclonal rabbit-anti-human glial fibrillary acidic protein (Dako Z0334), microglia/macrophages with 1:400 monoclonal mouse-anti-human CD68 (Dako M0876), and endothelial cells with 1:100 monoclonal mouse-anti-human CD31 (Dako JC70A). Antibodies were visualised using diaminobenzidine tetrahydrochloride (DAB), or Magenta Substrate Chromogen System GV925 when the black autometallography staining was obscured by the brown DAB. Oligodendrocytes were identified by their characteristic GFAP-negativity, cleared cytoplasm and contrast-enhanced nuclei.

### Laser ablation-inductively coupled plasma-mass spectrometry (LA-ICP-MS)

To confirm which metal autometallography was demonstrating, since autometallography can also detect inorganic silver and bismuth [29, 30], and to look for the presence of other potentially toxic elements, 7 μm paraffin sections of representative sections from patient PD2 were deparaffinised and subjected to LA-ICP-MS for mercury, silver, bismuth, aluminium, gold, cadmium, chromium, iron, nickel, lead and potassium. Analyses were carried out on a New Wave Research NWR-193 laser and a Teledyne Cetac LSX-213 G2+ laser hyphenated to an Agilent Technologies 7700x ICP-MS, with argon used as the carrier gas. LA-ICP-MS conditions were optimised on NIST 612 Trace Element in Glass CRM and the sample was ablated with a 50 μm spot size and a scan speed of 100 μm/s at a frequency of 20 Hz. The data were collated into a single image file using in-house developed software [31] and visualised using FIJI.

## Results

### Multifocal brain autometallography

The presence of autometallography-detected iHg was compared between the brains of people with PD, people without PD who had a known source of mercury exposure, and people without PD or a known source of mercury exposure, all of whom had iHg detected in locus ceruleus neurons.

#### Parkinson’s disease patients

**Fig 1:**
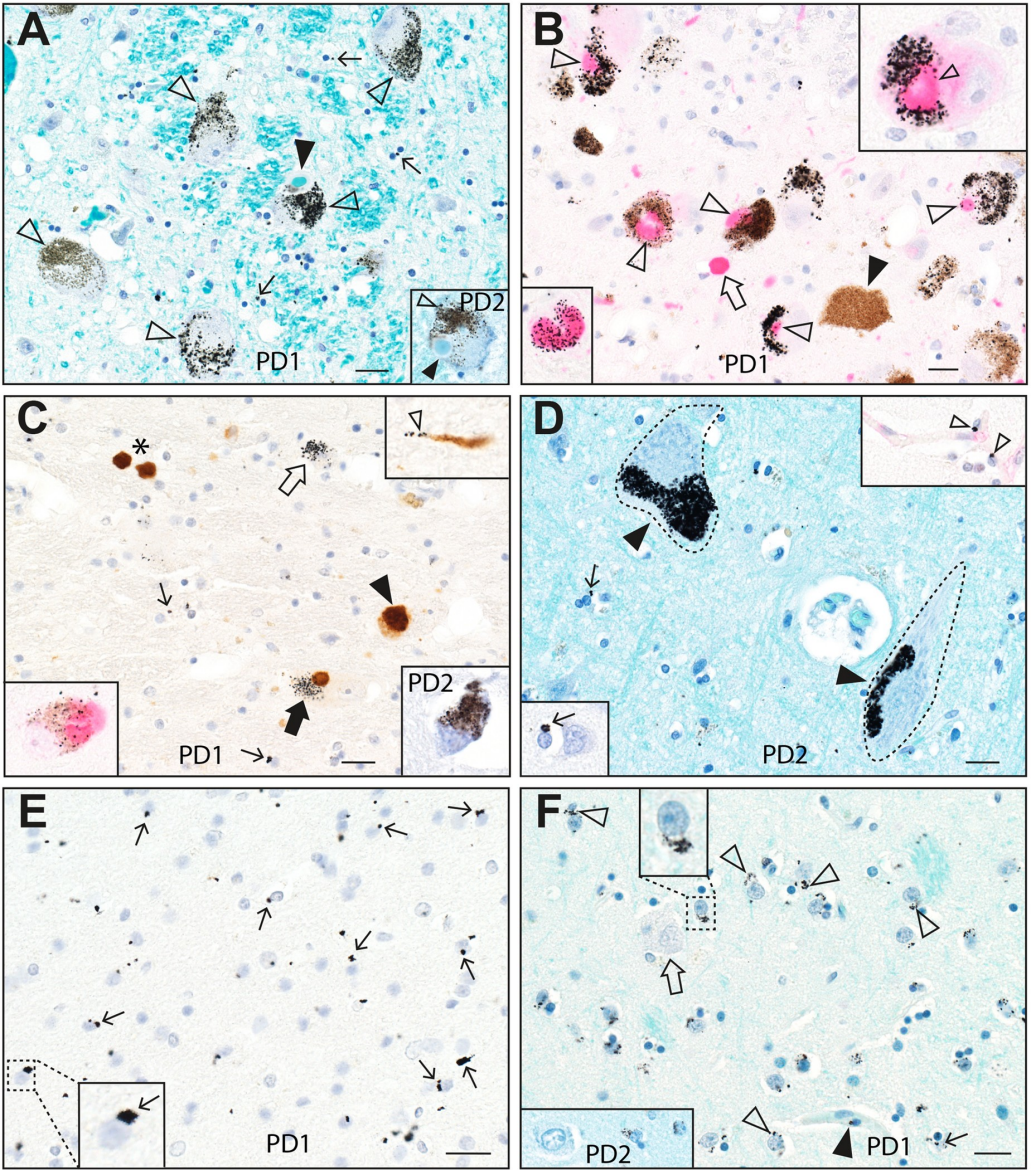
Mercury in the locus ceruleus, substantia nigra, cerebral cortex and striatum in Parkinson’s disease. (**A**) Black mercury grains (eg, open arrowheads) are present in the cytoplasm of most locus ceruleus neurons in PD1 (main image) and PD2 (inset). Lewy bodies with haloes (filled arrowheads) are seen in some mercury-containing neurons. Numerous oligodendrocytes have small mercury deposits (eg, arrows) adjacent to their nuclei. Autometallography/Luxol fast blue. (**B**) Magenta immunostaining of a-synuclein shows co-localisation of Lewy bodies (open arrowheads) with black-staining mercury in locus ceruleus neurons of PD1. An extra-neuronal a-synuclein aggregate (open arrow) has no associated mercury. No Lewy bodies are present in a locus ceruleus neuron not containing mercury (filled arrowhead). The right upper inset shows a nearby locus ceruleus neuron at higher magnification with Lewy body/mercury co-localisation. The left lower inset shows mercury within a Lewy body. Autometallography/a-synuclein Magenta/hematoxylin. (**C**) Brown DAB immunostaining of a-synuclein shows co-localisation of a Lewy body with mercury (filled arrow) in a remaining substantia nigra neuron of PD1. A nearby Lewy neurite (right upper inset) shows associated mercury grains (open arrowhead). Some intraneuronal (filled arrowhead) and extraneuronal (*) Lewy bodies do not appear to contain mercury, possibly because of masking by the dense brown DAB staining. Scattered oligodendrocytes have small mercury deposits (eg, thin arrows). Autometallography/a-synuclein DAB/hematoxylin. Left lower inset: Magenta a-synuclein staining shows black-staining mercury co-localised with a Lewy body in a nearby substantia nigra neuron (autometallography/a-synuclein Magenta/hematoxylin). Right lower inset: black-staining mercury is also present in a few remaining substantia nigra neurons in PD2 (autometallography/Luxol fast blue). (**D**) Dense mercury grains (filled arrowheads) are present in two corticomotoneuron cell bodies (dashed outlines) of PD2. Scattered oligodendrocytes (arrows) have small mercury deposits (one magnified in the left lower inset). Autometallography/Luxol fast blue. Two nearby pericytes contain mercury deposits (open arrowheads, right upper inset, autometallography/CD31/hematoxylin). (**E**) Numerous oligodendrocytes in the parietal white matter of PD1 have mercury deposits (eg, arrows) either attached to the nuclear membrane or adjacent to the nucleus. A magnified view is shown in the left lower inset. Autometallography/a-synuclein/hematoxylin. (**F**) Mercury grains (eg, open arrowheads) are present in the paranuclear region of medium-sized neurons in the putamen (one enlarged in the upper inset) of PD1. A large neuron (open arrow) contains no mercury. Small mercury deposits are present adjacent to nuclei of scattered oligodendrocytes (thin arrow) and pericytes (closed arrowhead). In the left lower inset, a similar distribution of mercury is seen in PD2 in medium-sized (right) but not large (left) neurons in the putamen. Autometallography/Luxol fast blue. PD: case numbers. Bars = 20 μm.

**Fig 2:**
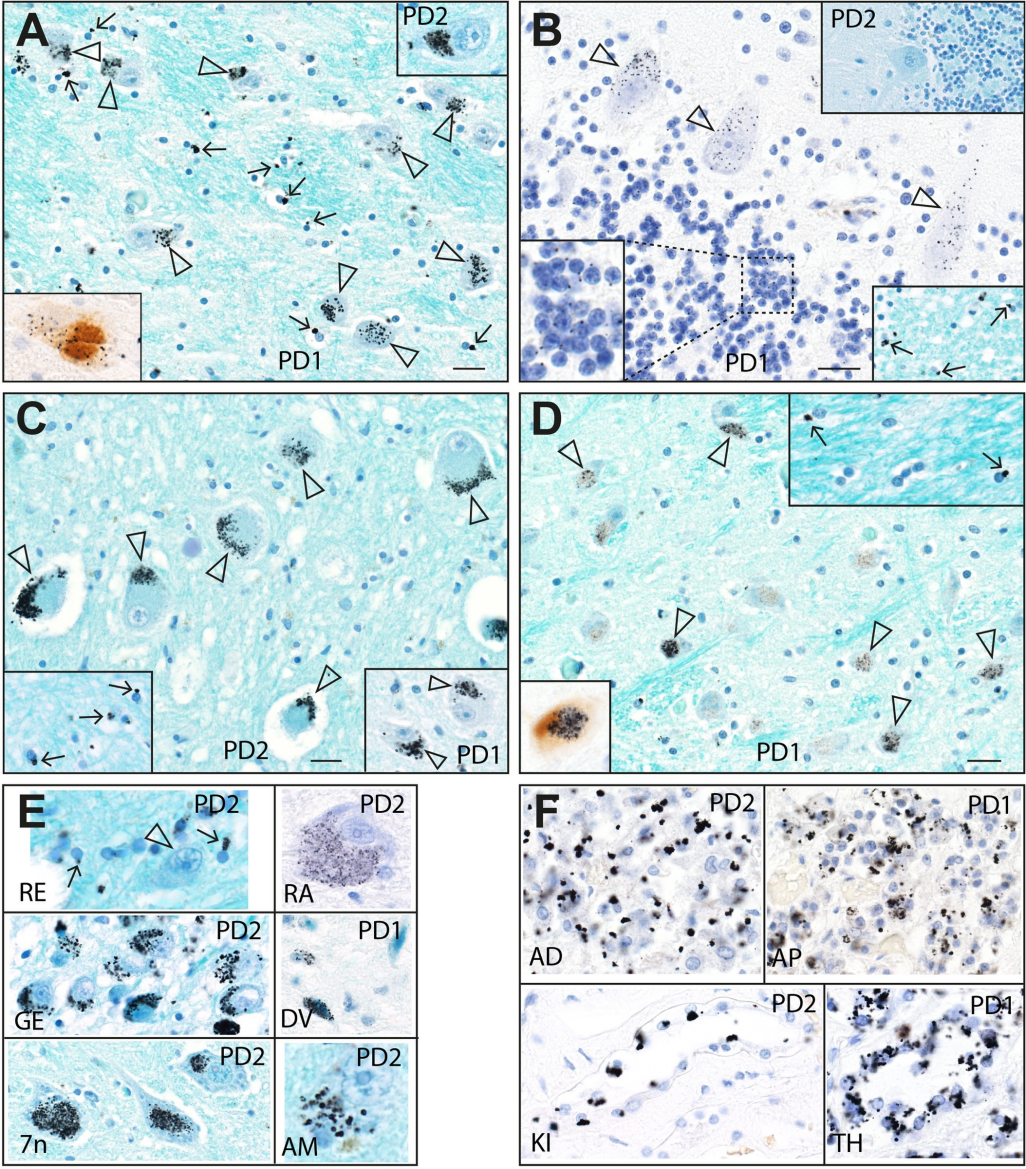
Mercury in the thalamus, cerebellum, other CNS regions, and non-CNS organs in Parkinson’s disease. (**A**) Black mercury grains are present in the cytoplasm of most thalamic neurons (eg, open arrowheads), in both PD1 (main image) and PD2 (right upper inset). Numerous oligodendrocytes have small mercury deposits (eg, arrows) adjacent to their nuclei. Autometallography/Luxol fast blue. A PD1 thalamic neuron with mercury co-localised with aggregated a-synuclein is seen (left lower inset, autometallography/a-synuclein DAB/hematoxylin). (**B**) Cerebellar Purkinje cells of PD1 contain small scattered cytoplasmic mercury grains (arrowheads). Cerebellar granule cells contain single paranuclear mercury grains (enlarged in the left lower inset). Scattered oligodendrocytes in the adjacent cerebellar white matter contain paranuclear mercury grains (eg, arrows, right lower inset). No mercury is present in the cerebellar cortex of PD2 (right upper inset). Autometallography/Luxol fast blue. (**C**) Black mercury grains (arrowheads) are present in the cytoplasm of cerebellar dentate neurons of PD2 (main image) and PD1 (right lower inset). Numerous oligodendrocytes in the nearby dentate internal white matter have paranuclear mercury deposits (arrows, left lower inset). Autometallography/Luxol fast blue. (**D**) Scattered neurons in the inferior olivary nucleus in the medulla oblongata of PD1 contain mercury grains (arrowheads). White matter internal to the olive contains oligodendrocytes with paranuclear mercury deposits (arrows, right upper inset). Autometallography/Luxol fast blue. Left lower inset: one nearby neuron in the inferior olivary nucleus has co-localised black mercury grains and brown aggregated a-synuclein (autometallography/a-synuclein DAB/hematoxylin). (**E**) Mercury grains are present in the juxtanuclear region of oligodendrocytes (arrows), but not of neurons (arrowhead), in the red nucleus (RE). Black mercury deposits are seen in the cytoplasm of neurons in the dorsal raphe nucleus (RA), lateral geniculate nucleus (GE), dorsal vagal nucleus (DV), the facial motor nucleus (7n) and the amygdala (AM). Autometallography/Luxol fast blue. (**F**) Mercury grains are present in cells of the adrenal medulla (AD), anterior pituitary (AP), kidney Henle loop (KI), and thyroid follicles (TH). Autometallography/hematoxylin. PD: case numbers. Bars = 20 μm.

*Neurons*. In the two people with PD, neurons containing cytoplasmic iHg were confirmed to be present in the locus ceruleus, and were also seen in some of the few surviving neurons of the substantia nigra compacta (referred to here as the substantia nigra), the frontal motor cortex (mostly corticomotoneurons/Betz cells), the striatum (medium-sized neurons in the caudate and putamen), thalamus, cerebellar cortex (Purkinje and granule cells), cerebellar dentate nucleus, lateral geniculate nucleus [32], dorsal raphe nucleus, cranial nerve motor nuclei 5, 6 and 7, dorsal vagal nucleus, nucleus ambiguus, and inferior olivary nucleus (**Figs 1 and 2 and Table 1**). The distribution of mercury between the two PD patients mostly overlapped, but some variability was seen: PD1 did not have mercury in cranial nerve motor nuclei or amygdala neurons, and PD2 did not have cerebellar neuronal mercury. Mercury was not seen in either patient in neurons of the globus pallidus, the subthalamic nucleus, the substantia nigra reticulata, or the basal nucleus.

**Table 1:** Mercury in neurons of people (1) with PD, (2) with mercury exposure but without PD, and (3) without known mercury exposure or PD.

	PD		Hg exposure		No PD, no known Hg exposure									
	72F	76F	24M	39M	59F	61M	61F	81F	86F	87M	89F	95F	98M	104F
Locus ceruleus	+	+	+	+	+	+	+	+	+	+	+	+	+	+
Substantia nigra	+	+	-	-	-	-	-	-	-	-	-	-	-	-
Cerebral cortex	+	+	+	-	-	-	-	-	-	-	-	-	-	-
Caudate/putamen	+	+	-	-	na	-	-	-	-	-	-	-	-	-
Thalamus	+	+	-	-	-	-	na	-	-	na	-	-	-	-
Subthalamic nucleus	-	-	-	-	na	-	-	-	-	-	na	-	-	-
Globus pallidus	-	-	-	-	na	-	-	-	-	-	-	-	-	-
Geniculate nucleus	+	+	-	+	-	+	-	-	-	-	-	-	-	-
Amygdala	-	+	-	-	-	-	-	-	-	-	-	-	-	na
Cerebellar cortex	+	-	-	-	-	-	-	-	-	-	-	-	-	-
Cerebellar dentate nucleus	+	+	+	+	-	+	-	-	-	-	-	-	-	-
Inferior olivary nucleus	+	na	-	+	-	-	-	-	-	-	-	-	-	-
Dorsal raphe nucleus	+	+	-	-	-	-	-	-	-	-	-	-	-	-
CN 5, 6, 7 motor nuclei	-	+	-	+	-	-	-	-	-	-	-	-	-	-
Dorsal vagal nucleus	+	+	-	-	-	-	-	-	-	-	-	-	+	-
Ambiguus nucleus	+	+	+	-	-	-	-	-	-	-	-	-	na	-

+ mercury present,—no mercury present, CN: cranial nerve, Hg: mercury, na: section not available, M: male, F: female, PD: Parkinson’s disease.

*Lewy bodies and neurites*. Mercury was co-localised with a-synuclein aggregates within some neurons or in the neuropil, especially in the locus ceruleus and substantia nigra (**Figs 1 and 2**). This co-localisation was not invariable, since some neurons contained mercury without a-synuclein aggregates, and some a-synuclein aggregates (particularly those free in the neuropil) did not contain mercury. Small mercury deposits were seen within some a-synuclein-positive Lewy neurites (**Fig 1**).

*Oligodendrocytes*. Juxta-nuclear mercury deposits were seen in multiple oligodendrocytes in both grey and white matter regions of the CNS (**Figs 1 and 2**). In the cerebral neocortex and hippocampus, the white matter oligodendrocytes most consistently affected were those in subcortical regions. The outflow tracts of grey matter regions where neurons contained mercury, such as the cerebellar dentate nucleus, contained many mercury-containing oligodendrocytes (**Fig 2)**.

*Other CNS cells*. Juxta-nuclear mercury deposits were seen in scattered pericytes (identified as cells adjacent to CD31-positive endothelial cells) throughout the CNS (**Fig 1**). No mercury was seen in astrocytes, microglia, or endothelial cells.

*Non-CNS cells*. In both PD patients, mercury was present in chromaffin cells of the adrenal medulla, kidney proximal tubules and thin Henle loops, and thyroid follicular cells (**Fig 2**). PD1 had mercury in anterior pituitary cells (the other PD case had no pituitary sample taken).

#### People with known sources of mercury exposure

**Fig 3:**
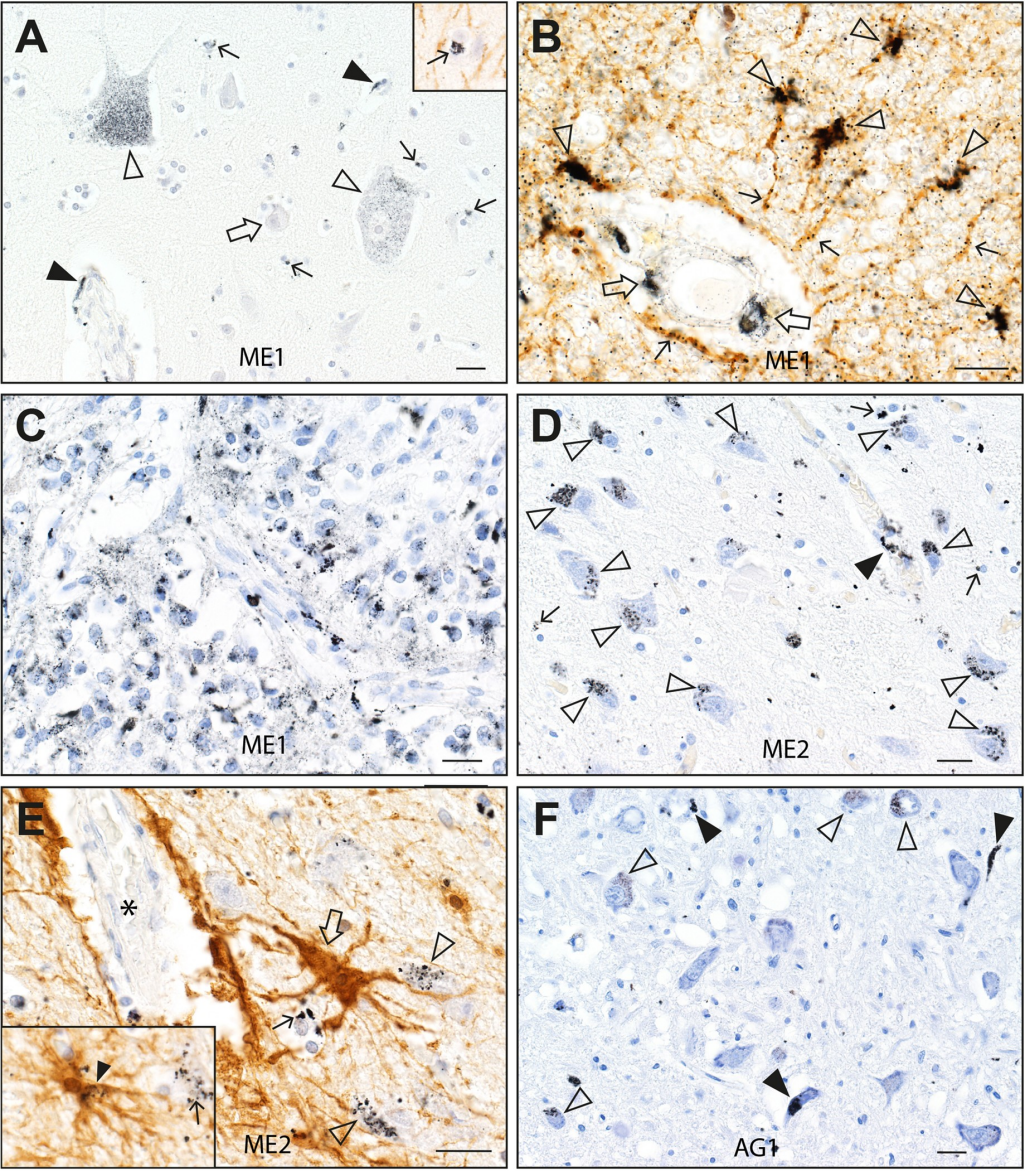
Mercury staining after exposure to mercury and in advanced age. (**A**) In ME1, black mercury grains are seen in the frontal motor cortex in two corticomotoneurons (open arrowheads), in scattered oligodendrocytes (eg, thin arrows), in endothelial cells (eg, closed arrowheads), but not in smaller cortical neurons (eg, open arrow). Autometallography/hematoxylin. Inset: a mercury-containing oligodendrocyte (arrow) is surrounded by GFAP-positive processes of interlaminar astrocytes (autometallography/GFAP DAB/hematoxylin). (**B**) In the frontal lobe white matter of ME1, mercury is present in endothelial cells (open arrows), and perivascular astrocyte cells bodies (eg, arrowheads) and processes (eg, thin arrows). Autometallography/GFAP DAB/hematoxylin. (**C)** Mercury is present in numerous pinealocytes in the pineal gland of ME1. Autometallography/hematoxylin. (**D**) In ME2, mercury grains are present in the cytoplasm of numerous lateral geniculate nucleus neurons (open arrowheads), scattered oligodendrocytes (eg, arrows), and in endothelial cells (filled arrowhead). Autometallography/hematoxylin. (**E**) In the lateral geniculate nucleus of ME2 a perivascular astrocyte (open arrow) has processes connecting a blood vessel (*) with mercury-containing neurons (open arrowheads) and oligodendrocytes (thin arrow). Inset: a nearby astrocyte process (closed arrowhead) contains mercury grains and connects with a mercury-containing oligodendrocyte (thin arrow). Autometallography/GFAP DAB/hematoxylin. (**F**) In the 95 years-old non-PD male control, some neurons (open arrowheads) and small blood vessels (filled arrowheads) in the dorsal vagal nucleus contain mercury. Autometallography/hematoxylin. ME: case numbers. Bars = 20 μm.

In the man exposed to metallic mercury (ME1), in addition to the locus ceruleus, neuronal iHg was seen in corticomotoneurons in the frontal cortex, cranial nerve motor nucleus 3 and 4, the cerebellar dentate nucleus, and in a few neurons in the nucleus ambiguus (**Fig 3**). Scattered oligodendrocytes in the frontal and occipital cortices and in the putamen had mercury deposits. Some endothelial cells in all CNS regions contained mercury. In the frontal white matter, perivascular astrocyte cell bodies and their processes connecting to the perivascular space contained mercury. Most pinealocytes contained mercury (**Fig 3**) (the pineal was not removed from other cases). Mercury was seen in the same cells of the adrenal medulla and kidney as above.

In the professional fisherman (ME2), in addition to the locus ceruleus, neuronal iHg was seen in neurons of the lateral geniculate nucleus [32], cuneate and gracile nuclei, cranial nerve motor nucleus 4, inferior olivary nucleus, medullary reticular formation and cerebellar dentate nucleus (**Fig 3**). Particulate mercury was seen adjacent to Purkinje cell bodies, possibly in Bergmann glia. A few scattered oligodendrocytes in most CNS regions contained mercury. Perivascular astrocytes containing mercury were in contact with lateral geniculate neurons and oligodendrocytes which both contained mercury (**Fig 3**). Mercury was seen in the same cells of the adrenal medulla, kidney and thyroid as above.

#### People without PD or known sources of mercury exposure

Among these 10 people, one 61 years-old male had iHg in lateral geniculate and cerebellar dentate neurons, and one 98 years-old male had iHg in a few neurons of the dorsal vagal nucleus (**Fig 3**). In the remaining eight people no brain regions apart from the locus ceruleus contained iHg. No oligodendrocyte iHg was seen in any of these ten brains. In this control group, iHg was present in eight of the nine kidney samples, six of the eight thyroid samples, and in all five pituitary samples taken.

#### Locus ceruleus-only autometallography

Autometallographic-detected iHg was present in 13 of 20 (65%) PD locus ceruleus samples and in 15 of 40 (63%) of the non-PD control locus ceruleus samples, an insignificant difference on contingency testing.

### LA-ICP-MS

**Fig 4:**
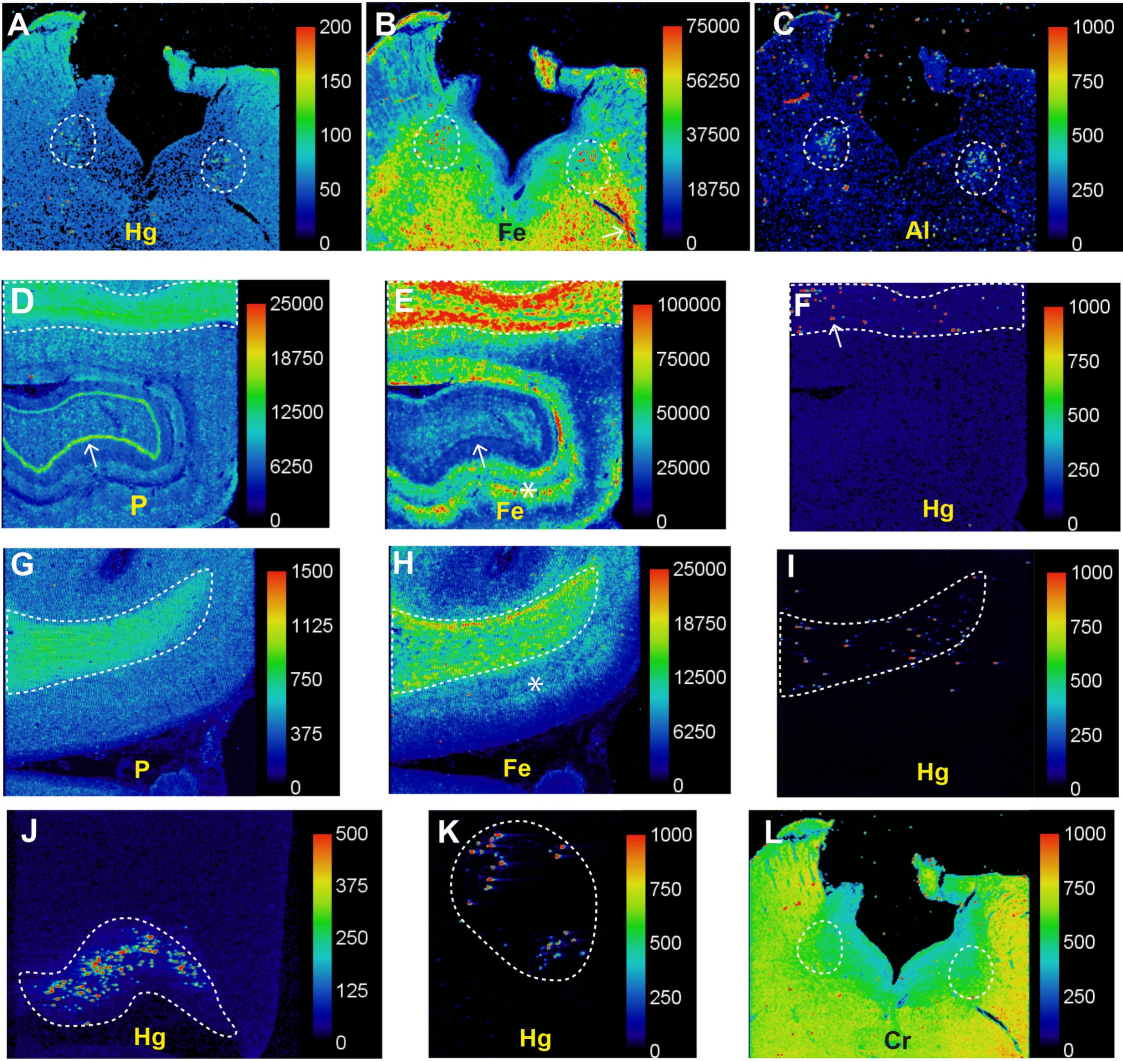
LA-ICP-MS in Parkinson’s disease (PD2). (**A**) Particulate mercury is seen in both locus ceruleus nuclei (outlined) where numerous neurons were autometallography-positive. (**B**) Particulate iron deposits are seen in both locus ceruleus nuclei (outlined) in the posterior pons. Linear deposits of iron in the pontine white matter (eg, arrow) are probably from red blood cells in blood vessels. (**C)** Particulate aluminium is seen in both locus ceruleus nuclei (outlined). (**D**) A normal high nuclear density shown by the phosphorus image is present in the hippocampal white matter (top, outlined) as well as in the dentate gyrus (arrow). (**E**) A large amount of iron is present in the hippocampal white matter (outlined), and in grey matter adjacent to the dentate gyrus (arrow, dark blue line). (**F**) Particulate mercury is seen in the hippocampal white matter (eg, arrow) where oligodendrocytes were autometallography-positive. (**G**) The normal high nuclear density of the frontal white matter (outlined) is shown in the phosphorus image. (**H**) The frontal white matter contains more iron than the frontal cortex, where most iron is in the deeper cortical layers (*) adjacent to the white matter. (**I**) Particulate mercury is present in the frontal white matter where oligodendrocytes were autometallography-positive. (**J**) Speckled mercury is present in the lateral geniculate nucleus where neurons were autometallography-positive. (**K**) Mercury is seen in pontine facial motor neurons, which were autometallography-positive. (**L**) Chromium is widespread in the posterior pons, without particular accumulation in the locus ceruleus (outlined). Scale = counts per second (proportional to abundance).

LA-ICP-MS of the posterior pons, lateral geniculate nucleus, facial motor nucleus, frontal lobe, hippocampus, and cerebellar hemisphere of PD2 showed mercury in regions where autometallography staining was positive, and did not detect mercury in autometallography-negative regions (**Figs 4 and S1**). Mercury was detected in regions containing large amounts of autometallography-detected intraneuronal iHg, such as the locus ceruleus, lateral geniculate neurons [32] and facial motor neurons, and in the frontal and hippocampal white matter where oligodendrocytes contained autometallography-detected iHg. The locus ceruleus also contained iron, aluminium and nickel, which were not seen in other regions. Iron was prominent in the hippocampal white matter and peri-dentate gyrus hippocampal grey matter, and present in the cerebral and pontine white matter, the deeper layers of the frontal cortex, and cerebellar subcortical white matter. Chromium was widespread in the posterior pons and hippocampus.

## Discussion

Key findings of this study are that people with PD who have been exposed to mercury (ie, with locus ceruleus mercury) had mercury within neurons and oligodendrocytes in regions of the brain known to be affected by PD, and had mercury associated with a-synuclein aggregates in Lewy bodies and neurites. This contrasts with people without PD (either those with or without known mercury exposure) who had previous mercury exposure (with mercury in locus ceruleus neurons) where mercury uptake was limited to a few brain sites and to non-CNS organs. People with and without PD appeared to have had similar exposures to mercury, judged by the presence of locus ceruleus mercury, suggesting that PD is more likely to result from a predisposition to take up toxic metals into the nervous system, rather than from environmental exposure to toxic metals such as mercury alone.

Synergistic interactions between metal toxins are increasingly being recognised [33, 34], so it is of interest that in a PD brain aluminium and nickel were seen together with mercury in the locus ceruleus. Mixtures of toxic metals, including mercury, cadmium, silver and lead, have previously been noted in the human locus ceruleus [7], indicating that uptake of multiple metal toxicants by the human brain is not unusual. Mercury itself can induce a-synuclein aggregation [20], but interactions between pesticides and metals can also accelerate the formation of a-synuclein fibrils in vitro [35] and so could be another factor underlying the formation of Lewy bodies and neurites in PD. Iron is present in large quantities in the human substantia nigra and locus ceruleus [36], and both mercury and iron were seen in white matter tracts in this PD case. The white matter iron, particularly high in the hippocampus, was probably within oligodendrocytes that are the predominant iron-containing cells in the brain [37, 38]. Iron has been suggested to play a role in PD [39] and in the synuclein disorder multiple system atrophy where oligodendrocytes contain a-synuclein inclusions [40]. Although interactions of brain iron and mercury are not described, the presence of both metals in PD oligodendrocytes, and in substantia nigra and locus ceruleus neurons, suggests synergy between these metals could make these cells susceptible to toxic damage.

**Fig 5:**
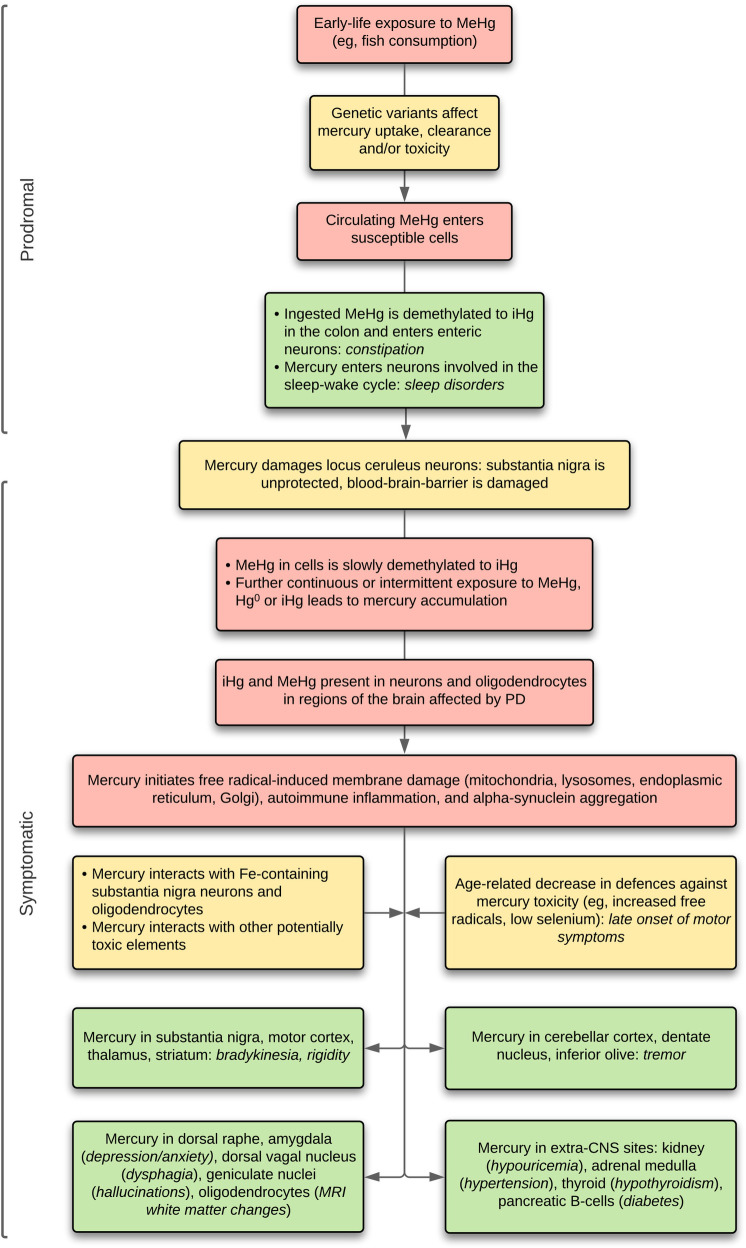
Hypothetical model of mercury-induced Parkinson’s disease. In this model, an early-life exposure to methylmercury (MeHg), aided by genetic susceptibility to brain mercury uptake, seeds susceptible cells, with later toxic effects modified by further genetic susceptibilities. During the prodromal period, mercury in colonic and sleep cycle-related neurons causes constipation and sleep disorders. Early uptake of mercury by the locus ceruleus places the substantia nigra at risk of toxicant damage, and permits toxicants to pass through the blood-brain-barrier. Inorganic mercury (iHg) slowly accumulates in neurons and oligodendrocytes, both from demethylation of methylmercury and from further exposures to mercury. When cellular mercury concentrations reach a critical level in neurons and oligodendrocytes, aided by synergistic effects in iron-containing cells, interactions with other toxicants, and decreasing aging-related natural defenses, mercury damages membranous organelles in these cells and promotes a-synuclein aggregation. This results in symptomatic PD, with motor, non-motor, and non-CNS associated symptoms. Hg°: mercury vapor.

Based on the distribution of mercury within the brain, a hypothetical model of toxicant-induced PD can be constructed, with the metal playing a part in both the prodromal period and in the later motor and non-motor manifestations, as outlined in **Fig 5** and detailed below.

*Prodromal stage*. Early life exposure to methylmercury, probably from fish consumption, could result in mercury entering susceptible cells via methionine receptors [13]. The American Academy of Pediatrics states that fish can be added to a baby’s food within a few months of starting solid foods, and the USA Food and Drug Administration recommends up to three servings of fish per week from the age of two years, with avoidance of high-mercury fish such as shark (www.FDA.gov/fishadvice). However, in many countries the labelling fish is inadequate and cheaper fish often contain shark meat. Two prominent PD prodromal symptoms, constipation and disordered sleep, could be related to mercury exposure (**Fig 5**). This is because ingested methylmercury is demethylated in the colon to inorganic mercury [13] which can then be absorbed and enter enteric neurons to reduce colon mobility and cause constipation (no colonic samples were available from our samples to look for mercury in enteric neurons). Second, mercury was found in pinealocytes in the man exposed to metallic mercury, suggesting that changes in melatonin secretion, important for the sleep-wake cycle, could be present after mercury exposure. In addition, neurons of the suprachiasmatic nucleus, which controls the timing of the sleep-wake cycle, selectively take up bismuth [41], which has the same tissue distribution as mercury [42]. The suprachiasmatic nucleus is often damaged during autopsy brain removal and could not be identified in our samples.

*Progression to symptomatic PD*. Several factors could increase the chance for mercury to promote progression of PD prodromal symptoms to later motor disorders (**Fig 5**). (**1**) The ability of the body to deal with the toxic effects of mercury depends on the actions of many genes [14, 43]. Polymorphisms in these genes may affect the ability of cells to control mercury entry, toxicity or elimination and make these cells more susceptible to mercury-induced damage. (**2**) The locus ceruleus is damaged early in most cases of PD [44, 45]. Mercury in locus ceruleus neurons would reduce noradrenaline output and impair the integrity of blood-brain-barrier [46], allowing easier access to the CNS to circulating toxicants. Furthermore, the locus ceruleus protects the substantia nigra from damage [44, 45, 47] so any toxicants within the substantia nigra would be more active if locus ceruleus neurons were not producing noradrenaline. Damage to the locus ceruleus could also contribute to non-motor PD symptoms such as autonomic disturbances, sleep disorders, depression, cognitive difficulties, and hyposmia [45, 48]. (**3**) Repeated or continuous exposure to environmental sources of mercury would increase the level of mercury within cells, until a tipping point was reach in later age. Common sources of mercury are from eating mercury-contaminated fish, certain occupations, and dental amalgam fillings [13, 14]. Of note, the prevalence of inorganic mercury in human cells has been found to increase during aging [16] which could contribute to the increasing PD symptoms on aging. (**4**) Decreased defences to mercury neurotoxicity because of aging, for example from an increased production of reactive oxygen species [49] or from reduced levels of selenium [50], could also contribute to the appearance of PD symptoms in later life. This is relevant to the frequent finding of Lewy bodies in normal elderly subjects, which may represent preclinical PD [51], since mercury can persist in substantia nigra neurons many years after initial exposure without causing symptoms [52].

**Fig 6:**
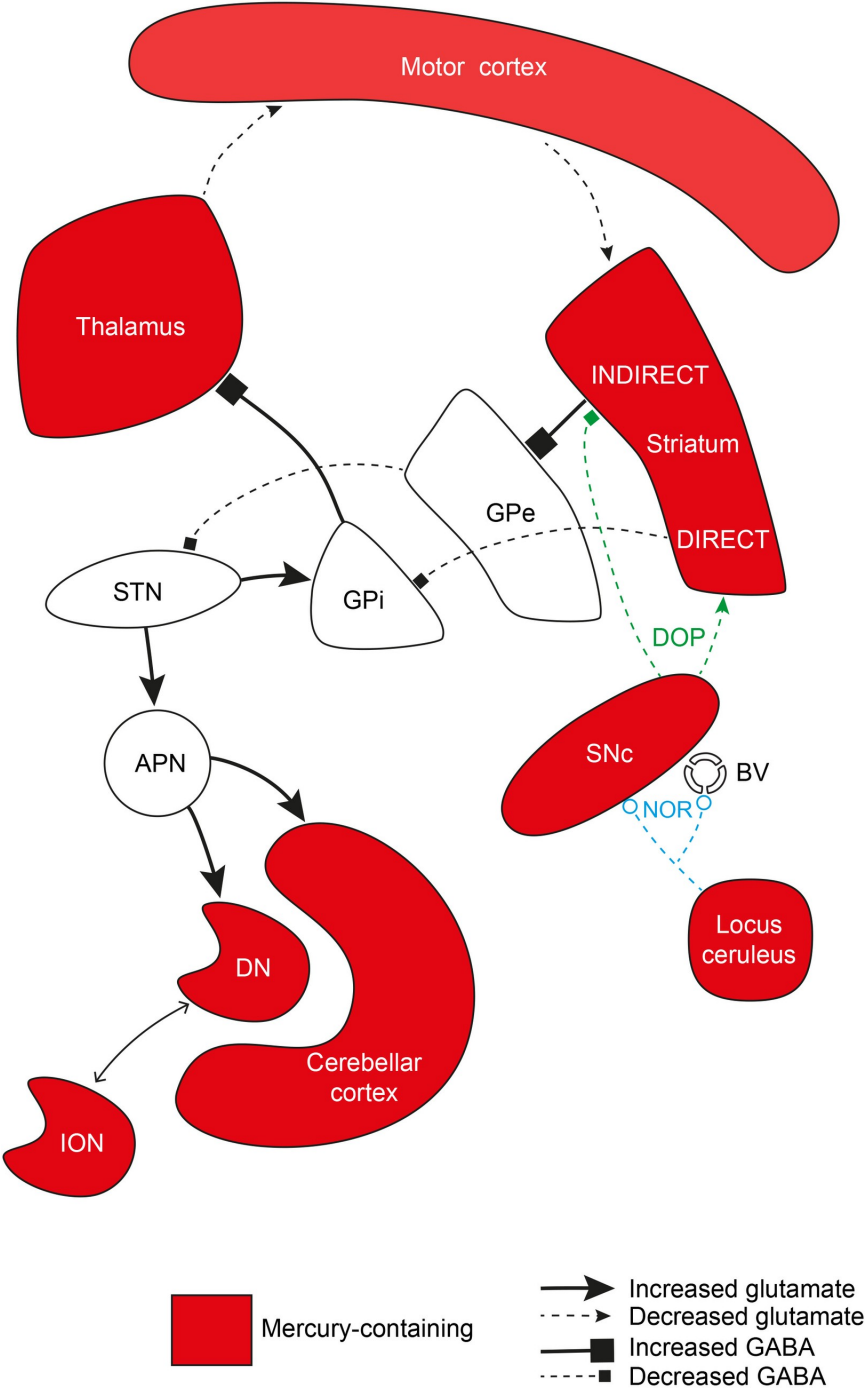
Mercury in neurons that could contribute to bradykinesia, rigidity and tremor. Regions of the brain implicated in PD motor disorders whose neurons contain mercury are shown in red. The classical pathway of PD motor pathophysiology is included in the upper half of the diagram. Neurons in the substantia nigra compacta (SNc), striatum, thalamus and motor cortex contain mercury, but not those in the subthalamic nucleus (STN), or the globus pallidus interna (GPi) and externa (GPe). Mercury in the locus ceruleus could decrease noradrenaline output to the substantia nigra, leaving it more susceptible to toxicants, and to blood vessels, making the blood-brain-barrier more permeable to circulating toxicants. Mercury in neurons of the cerebellar cortex, dentate nucleus (DN) and inferior olivary nucleus could underlie types of tremor in PD (lower left in diagram). Also shown (bottom left) is a stimulatory pathway from the subthalamic nucleus via the anterior pontine nuclei to the cerebellar cortex and dentate nucleus (both containing mercury), that could cause a resting tremor. BV: blood vessel, DIRECT: direct pathway, INDIRECT: indirect pathway, DOP: dopamine, GABA: gamma-aminobutyric acid, NOR: noradrenaline.

*Bradykinesia and rigidity*. Bradykinesia and rigidity are major motor features of PD [53]. In the classical model of motor circuit pathophysiology, bradykinesia and rigidity occur when decreased dopamine from the substantia nigra has opposing effects on the striatal direct and indirect pathways, leading to suppressed globus pallidus externa firing with consequently increased subthalamic nucleus activity, as well as increased globus pallidus interna-mediated thalamic inhibition [54] (**Fig 6**). In our PD patients, neurons containing mercury were prominent in the substantia nigra, motor cortex, and thalamus, all regions with a decreased neuronal output in the classical model. The mercury content of corticomotoneurons was high, which may be because of a trans-synaptic passage of mercury from the adrenal medulla [55] or kidney [56], both of which contained mercury in our patients. This may be important since mercury in corticomotoneurons could affect the hyperdirect pathway of motor control [57]. Mercury was seen in many medium-sized neurons in the striatum, which are implicated in the pathophysiology of PD [58]. The subthalamic nucleus and globus pallidus, regions affected by changes to striatal neurons in the classical model, did not have mercury-containing neurons. This distribution suggests mercury could contribute to bradykinesia and rigidity in idiopathic PD by disrupting basal ganglia/thalamic/motor cortex communications.

*Tremor*. A resting tremor is characteristic of PD, but about a quarter of people with PD do not have a tremor, and of those who do, most have a mixed resting and action tremor, while a few have an action tremor only [59]. The pathogenesis of tremor in PD is unclear, and is not readily explained by the classical pathophysiology model, with possible sources being the basal ganglia, thalamus, or cerebellum. Recent findings that a subthalamic nucleus/anterior pons/cerebellar cortex pathway can generate a resting tremor suggests a key role for the cerebellum in the genesis of PD tremors [60] (**Fig 6**). It has long been known that the most frequent sign of occupational mercury exposure is an action tremor, probably from mercury in the basal ganglia or cerebellum [61]. The cerebellar cortex has also been implicated in action tremors in post mortem studies while others have suggested the inferior olivary nucleus could be involved [62]. In our PD cases, mercury was present variably in cerebellar Purkinje and granule neurons, and in neurons of the cerebellar dentate and inferior olivary nuclei (**Fig 6**). This variability in toxicant distribution could explain the differences in tremor type between PD patients.

*Non-motor CNS symptoms*. Several non-motor PD CNS symptoms could be triggered by a toxicant such as mercury. (**1**) The incidence of dementia in people with PD is fourfold higher than in the general population, with the average time to dementia after PD diagnosis being about 10 years [63, 64]. One possible contributor to PD dementia could be widespread damage to oligodendrocytes from contained mercury, as seen in our PD cases. Oligodendrocytes contain iron [38], and a large amount of iron was present in hippocampal white matter where oligodendrocytes contained mercury, so a synergistic effect of these two metals could reduce oligodendrocyte function in this region that is vital for memory. Oligodendrocytes have previously been implicated in both Alzheimer’s disease [65] and in a-synucleinopathies [66], and so may be involved in the combination of a-synuclein and Alzheimer pathology often present in PD dementia [63]. Non-myelinating oligodendrocytes in PD that contain a-synuclein inclusions are damaged late in the disease [67], when dementia usually occurs. Widespread damage to oligodendrocytes could also be the basis for the white matter changes seen on MRI in PD [68], and it has been suggested that white matter impairment precedes neuronal loss in PD [69]. (**2**) Visual hallucinations are frequent in PD, and can be a harbinger of future dementia [64]. Damage to the lateral geniculate nucleus, with disturbances of the visual pathway, may be triggered by mercury in geniculate neurons [32]. Both of our PD cases had marked uptake of mercury into lateral geniculate neurons. (**3**) Depression and anxiety are common in PD [1]. Mercury in serotonergic brain stem raphe neurons could contribute to depression, and mercury in amygdala neurons may play a part in anxiety. (**4**) It is unclear why pain should accompany PD [1], but mercury was present in thalamic neurons in our PD cases and is also taken up by human posterior root ganglia neurons [52], so either central or peripheral regions of the pain pathway could be disturbed after mercury exposure. (**5**) Both retinal thinning on optical coherence tomography [70–72] and retinal pathology [73] have been noted in people with PD. This may relate to mercury being found commonly in the human retinal pigment epithelium and choriocapillaris [74], as well as mercury being present in retinal ganglion cells, pigment epithelium and endothelial cells of mercury-exposed mice [75] and primates [76].

*Non-CNS associated symptoms*. In our PD cases, mercury was present in cells of the kidney, thyroid, adrenal medulla and anterior pituitary. This could explain the presence of some extra-CNS disorders associated with PD. Chronic kidney disease [23] and hypertension [77, 78] appear to be more common in PD, and mercury in both the renal cortex and medulla can be found in older adults [79], including our PD patients. Kidney mercury could be responsible for the low levels of uric acid seen in PD [80], since chronic exposure to heavy metals can increase urate secretion [81]. Mercury can be taken up by thyroid follicular cells and could be responsible for the thyroid disorders that are found in about 10% of PD patients [21, 82]. Mercury in the adrenal medulla may contribute to hypertension by raising noradrenaline levels [83] and therefore raise blood pressure in PD patients [77, 78]. Pancreatic samples were not available for our PD cases, but mercury can be taken up by human pancreatic insulin-producing beta cells [16], so mercury exposure could contribute to the increased incidence of type 2 diabetes in PD [22, 84].

This study has several limitations. (**1**) No detailed clinical information was available from these forensic cases, so we were not able to match neurological signs and symptoms with the distribution of mercury in the brain. (**2**) No extensive data on environmental exposure to mercury, such as occupations, fish consumption, and numbers of dental amalgam fillings [85], were available. (**3**) We limited the study to people with evidence of previous mercury exposure (ie, with mercury in the locus ceruleus) to compare the multifocal distribution of mercury in the brains of people with or without PD. This reduced the number of people with PD available for study. Future multimodal elemental studies of larger numbers of PD post mortem brains, both with and without histochemical evidence of mercury exposure, are likely to give further insights into the role of toxic metals in PD. (**4**) In many people with PD a long latent period between the last toxicant exposure and the time of death is likely, and clearing of toxicants from cells over time, for example by the glymphatic pathway [32], is to be expected. It is therefore unlikely that in all PD patients, even those in whom toxicants played a role, brain toxicants will readily be identified using current techniques based on post mortem samples. Future in vivo multi-elemental imaging studies of younger people with PD, or those with rapidly progressive disease, could give a more comprehensive picture of toxicant distribution in PD brains. (**5**) The numbers of surviving substantia nigra neurons in our PD patients were too small to confirm the presence of mercury, or to look for other neuronal toxic elements, with LA-ICP-MS. However, others have previously shown the presence of mercury and other metals in neuromelanin from the substantia nigra [86]. (**6**) It is rare to obtain human post mortem brain samples that have been subjected to modern elemental analysis from people with a known exposure to mercury, and our two mercury-exposure cases were younger than the PD cases, and of different gender. However, we think these cases are worth including since they do suggest that people without PD handle mercury differently, with a restricted brain uptake. Furthermore, PD has a long prodromal period, so it is likely that the initial toxicant exposure occurs years before the clinical manifestations of PD appear, when the patient is younger. (**7**) The mean age of our control brain samples (82 years) was higher than that of the PD cases (74 years). However, since mercury accumulates with age in human cells [16], the relative lack of mercury in this older control group is a further indication of the restricted distribution of mercury in most non-PD cases.

In conclusion, mercury can be found in neurons and oligodendrocytes in regions of the brain that are affected by PD, and mercury often co-localises with the aggregated a-synuclein found in Lewy bodies and neurites. Although post mortem tissue studies cannot provide a direct link between toxicants and PD pathogenesis, the varied toxic mechanisms of mercury make it a candidate for an environmental toxicant that could play a role in the disease, probably in conjunction with genetic susceptibilities to metal uptake, elimination, or toxicity, and synergies with other potentially toxic elements. The development of future in vivo bio-elemental imaging techniques will be needed to further study the role of toxic elements in the prodromal and early symptomatic phases of the disease. In the longer term, it will of interest to see if current attempts to decrease the burning of fossil fuels, a major contributor to rising atmospheric mercury levels and related mercury pollution of fish, will result in a future decrease in the incidence of idiopathic PD.

## Supporting information

S1 Fig All LA-ICP-MS images (patient PD2). The labels indicate the outlined regions. Phosphorus images (top row) indicate the nuclear density of the tissues. (**A**) Particulate metals detected in the autometallography-positive locus ceruleus are mercury, iron, aluminium, and nickel. Iron in prominent in the pontine white matter. Chromium is widespread in the posterior pons. (**B**) Speckled mercury is present in the lateral geniculate nucleus, where neurons were autometallography-positive. Iron is seen in the adjacent white matter. (**C)** Mercury is present in the region of facial motor neurons. (**D**) Particulate mercury is present in the frontal white matter, which contains more iron than the cortex. (**E**) Particulate mercury is seen in the hippocampal white matter, which contains a large amount of iron. Chromium is widespread in the hippocampus. (**F**) No mercury is seen in the cerebellar cortex or white matter, which were both autometallography-negative in this patient. Iron is present in the cerebellar subcortical white matter. No significant amounts of cadmium, lead, bismuth, silver or gold are detected in any sections. Scale = counts per second (proportional to abundance).
(TIF)
