# Supplementary figures and images for: Mercury is present in neurons and oligodendrocytes in regions of the brain affected by Parkinson’s disease and co-localises with Lewy bodies
Source: PLoS One. 2022 Jan 11;17(1):e0262464. doi: 10.1371/journal.pone.0262464 (PMC8752015; doi:10.1371/journal.pone.0262464)

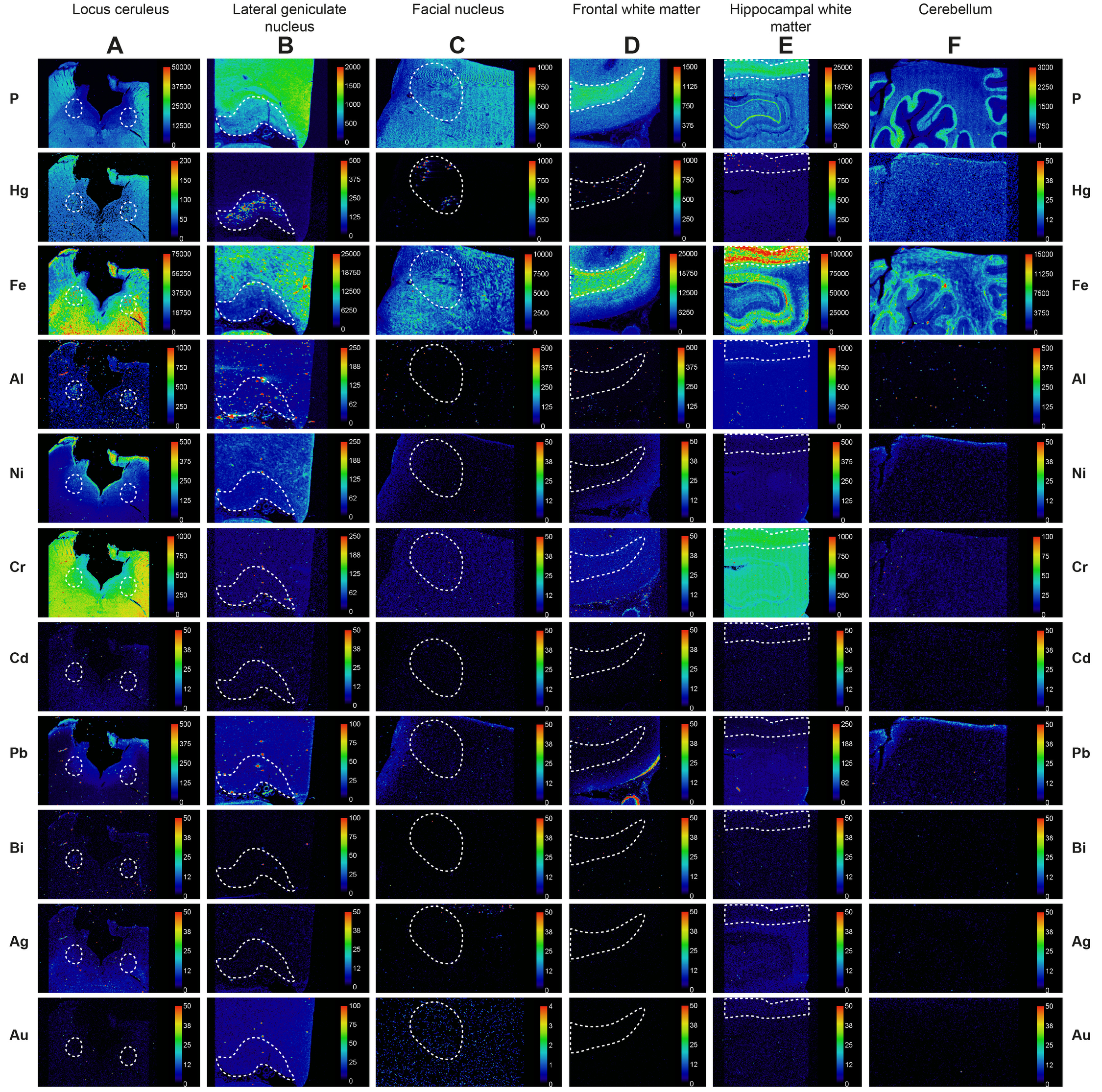

Supplement: S1 Fig — The labels indicate the outlined regions. Phosphorus images (top row) indicate the nuclear density of the tissues. (A) Particulate metals detected in the autometallography-positive locus ceruleus are mercury, iron, aluminium, and nickel. Iron in prominent in the pontine white matter. Chromium is widespread in the posterior pons. (B) Speckled mercury is present in the lateral geniculate nucleus, where neurons were autometallography-positive. Iron is seen in the adjacent white matter. (C) Mercury is present in the region of facial motor neurons. (D) Particulate mercury is present in the frontal white matter, which contains more iron than the cortex. (E) Particulate mercury is seen in the hippocampal white matter, which contains a large amount of iron. Chromium is widespread in the hippocampus. (F) No mercury is seen in the cerebellar cortex or white matter, which were both autometallography-negative in this patient. Iron is present in the cerebellar subcortical white matter. No significant amounts of cadmium, lead, bismuth, silver or gold are detected in any sections. Scale = counts per second (proportional to abundance). (TIF) [file pone.0262464.s001.tif]
